# Profiles of pregnant women encountering motor vehicle crashes in Taiwan, 2008–2017

**DOI:** 10.1186/s40621-023-00478-x

**Published:** 2023-12-19

**Authors:** Ya-Hui Chang, Yu-Wen Chien, Chiung-Hsin Chang, Ping-Ling Chen, Tsung-Hsueh Lu, Chang-Ta Chiu, Chung-Yi Li

**Affiliations:** 1 Department of Public Health, College of Medicine, National Cheng Kung University, Tainan, Taiwan; 2 Department of Surgery, Massachusetts General Hospital, Boston, MA USA; 3 Department of Obstetrics and Gynecology, National Cheng Kung University Hospital, College of Medicine, National Cheng Kung University, Tainan, Taiwan; 4 Graduate Institute of Injury Prevention and Control, College of Public Health, Taipei Medical University, Taipei, Taiwan; 5 Department of Dentistry, An Nan Hospital, China Medical University, Tainan, Taiwan; 6 Department of Public Health, College of Public Health, China Medical University, Taichung, Taiwan; 7 Department of Healthcare Administration, College of Medical and Health Science, Asia University, Taichung, Taiwan

**Keywords:** Pregnancy, Taiwan, Traffic collision, Trimester, Type of vehicle

## Abstract

**Background:**

Understanding demographic profiles is essential to the assessment of health burden imposed by motor vehicle crashes (MVCs) on pregnant women. However, Asian studies that have examined it are lacking. The study aimed to describe the demographic characteristics and prevalence of MVCs involving pregnant women in Taiwan.

**Methods:**

A cross-sectional study conducted by the Taiwan Birth Notification dataset from 2008 to 2017 was linked with the police-reported traffic collision registry to identify pregnant women involved in MVCs. The pregnant women were categorized according to their gestational age, age at delivery, the role of road user (driver, passenger, or pedestrian), and vehicle types (car, two-wheeled motor vehicle, or others). A chi-square test was performed for the significance test.

**Results:**

A total of 22,134 (1.13%) pregnant women were involved in MVCs in the study period. Two-wheeled motor vehicle (47.9%) and driver (81.4%) were the mainly reported vehicle type and road user at the crash scenes, respectively. The majority of MVCs occurred in pregnant women aged 28–34 years. The number of MVCs rapidly declined after 37 weeks of gestation, especially two-wheeled motor vehicle or car crashes. However, the number of pedestrian victims climbed up during the third trimester.

**Conclusion:**

Pregnant women are susceptible to MVCs regardless of their gestational age, role of a road user, or type of vehicle. The findings of this study emphasize the need for increased awareness of traffic collision prevention among pregnant women aged 28–34. In addition, improving pedestrian safety is essential for the reduction of pregnant victims.

**Supplementary Information:**

The online version contains supplementary material available at 10.1186/s40621-023-00478-x.

## Background

Motor vehicle crashes (MVCs) are the common cause of reported trauma during pregnancy (Mendez-Figueroa et al. 2013). Pregnant women who encounter MVCs may suffer from serious maternal adverse outcomes (Chang et al. 2023), and fetuses are at a potential risk of injury (Vladutiu et al. 2013a). Moreover, the burden associated with healthcare costs and resource use after the maternal injury is substantial (Owens et al. 2008; Periyanayagam & Crandall 2014). Studies describing the consequences of road traffic collisions were mostly performed in Western countries (Mayou & Bryant 2003). Consequently, few studies have addressed the characteristics of pregnant women involved in MVC in Asian countries. Even a study conducted in Turkey showed that 73% of patients were involved in car accidents (Soysal et al. 2021). This can lead to misunderstandings regarding the appropriate target population, mainly because of notable differences in road conditions and the type of vehicle used between different countries. For example, the number of motorcycles in Taiwan is extremely high (Lam et al. 2019), and in the United States, passenger car is the predominant vehicle type in traffic crashes (National Center for Statistics and Analysis, 2023).

Information on the demographic characteristics of pregnant women who encounter MVCs is essential to understanding of the magnitude of health burden imposed by MVCs on pregnant women. The aim of this study was to describe the profile of women who encountered MVCs during pregnancy in Taiwan. Specifically, this study addressed the following questions: which age group contributes the highest proportion among pregnant MVCs victims, during which trimester do most pregnant women experience MVCs, and which type of vehicle is associated with the highest number of MVCs involving pregnant women.

## Methods

The study encompassed a nationwide population of 1,961,205 delivered mothers aged 18–50 who experienced MVCs during pregnancy in Taiwan from 2008 to 2017. The data for this research were obtained from the Taiwan Birth Notification (BN) and the police-reported traffic collision registry. The BN contains information on the birthplace, demographic characteristics, birth attributes (such as birthweight and gestational age), and other relevant details of mothers (Hsu et al. 2023). In Taiwan, all live births or stillbirths (longer than 20 weeks or weight equal/over 500 g) should be registered within seven days of their occurrence. The traffic collision registry, administered by the National Police Agency of Taiwan, records comprehensive reports on traffic collisions, including detailed information on crash scenes and individuals involved in MVCs. Trained police officers visit crash locations to gather data (Chang et al. 2020, 2022). The study received approval from the Institutional Review Boards of National Cheng Kung University Hospital (No. B-ER-109-88) in Taiwan.

To identify pregnant women involved in MVCs during their pregnancy, the BN dataset from 2008 to 2017 was linked to the traffic collision registry data from 2007 to 2017. The link was established according to the encrypted personal identification number of each study subject. The number and prevalence of MVCs were categorized by vehicle type (car; two-wheeled motor vehicle: including motorcycle, scooter or mopeds; others: including the pedestrian because the pedestrian did not belong to any vehicle at scene, as well as other vehicles such as buses, trucks, and bicycles) or by role of road users (driver; passenger; pedestrian) from the police officers and recorded in the victim profile from collision data. Additionally, the geographical area and level of urbanization were determined according to the city district/township of residence. The geographical area was divided into four clusters (north; central; south; and east, as shown in Additional file 1: Fig. A3). The urbanization levels of Taiwan’s 368 cities and townships were established using the methodology by Liu et al. (2006). This method categorized all of Taiwan’s city districts and townships into seven distinct levels of urbanization based on a range of indicators, including population density, proportion of residents with college or higher education, percentage of older people (aged over 65), the proportion of workforce engaged in agriculture, and the number of physicians per 100,000 people. The prevalence of MVCs was examined by trimester.

In statistical analysis, the study firstly presented the number and prevalence of overall MVCs, and MVCs associated with specific vehicle types or roles of road users. The distributions of MVC event numbers related to specific vehicle types or roles of road users were compared in terms of maternal age, calendar year, geographical area of residence, and level of urbanization by the Pearson's chi-square test. However, the chi-square is very sensitive to sample size. With a large enough sample, statistically significantly does not necessarily mean “meaningful.” In addition, the three trimesters were compared according to the proportion of MVC events categorized by vehicle type and role of road users. The number of MVCs was graphically illustrated by gestational week or maternal age and further stratified according to vehicle type and role of road users. All these analyses were conducted using SAS (version 9.4).

## Results

A total of 1,961,205 mothers who gave birth between 2008 and 2017 were included in the study. Among them, 21,875 pregnant women experienced 22,134 maternal MVCs during pregnancy. In other words, 21,618 of pregnant women experienced only one MVC, 255 pregnant women encountered two MVCs, and 2 pregnant women experienced three MVCs during their pregnancy period. Table 1 illustrates that 1.13% of pregnant women encountered MVCs, showing a significantly high prevalence was observed among mothers aged 18–24 (*p *< 0.001). Furthermore, the prevalence of MVCs significantly increased in recent years (2016–17, *p *< 0.001), and the figure for this period was nearly twofold those for 2008–2009 (1.4% vs. 0.8%). Pregnant women residing in the east or rural areas had a significantly higher prevalence of MVCs during pregnancy than those in other locations (*p *< 0.001).

**Table 1:** Prevalence of motor vehicle crashes (MVCs) among women who gave births between 2008 and 2017 in Taiwan, according to vehicle type

	No. of deliveries	MVC during pregnancy		
		Yes	Prevalence	(95%CI)
Total	1,961,205	22,134	1.13	(1.11–1.14)
*Maternal age at delivery (years)*				
18–24	179,072	3203	1.79	(1.73–1.85)
25–29	515,943	5927	1.15	(1.12–1.18)
30–34	796,206	8146	1.02	(1.00–1.05)
35–39	400,020	4105	1.03	(1.00–1.06)
40 +	69,964	753	1.08	(1.00–1.16)
Mean ± SD	31.12 ± 4.77	30.41 ± 5.20		
*Calendar year of delivery*				
2008–2009	380,380	3097	0.81	(0.79–0.84)
2010–2011	355,896	3456	0.97	(0.94–1.00)
2012–2013	418,540	4514	1.08	(1.05–1.11)
2014–2015	414,356	5459	1.32	(1.28–1.35)
2016–2017	392,033	5608	1.43	(1.39–1.47)
*Geographical area of residence*^a^				
North	945,611	7792	0.82	(0.81–0.84)
Central	491,868	7061	1.44	(1.40–1.47)
South	475,508	6588	1.39	(1.35–1.42)
East	40,647	624	1.54	(1.42–1.66)
*Urbanization of residence *^a^				
Urban	672,447	7184	1.07	(1.04–1.09)
Satellite	765,007	8072	1.06	(1.03–1.08)
Rural	503,766	6595	1.31	(1.28–1.34)

^a^The inconsistency observed between the total population number and the sum of individual variable populations can be attributed to missing information

Table 2 provides a breakdown of MVCs by vehicle type at the scene. Two-wheeled motor vehicle crashes constituted the majority (47.9%), followed by cars (33.1%) and other vehicle types (19.0%). Throughout the study period, a consistently high proportion of two-wheeled motor vehicle crashes were observed, and no urban–rural disparity was found. The highest proportion of two-wheeled motor vehicle crashes involved mothers aged 18–24 (55.3%), and the highest proportion of car crashes involved mothers over 40 years old (45.8%). Furthermore, significant regional variation in the percentages of different types of vehicle collisions was observed. In the southern region, two-wheeled motor vehicle crashes accounted for the highest proportion, reaching 50.8%. However, in the eastern region, car crashes had the highest proportion, accounting for 41.2%. Table 2 indicates that the majority of mothers involved in MVCs were drivers (81.4%), followed by passengers (15.9%) and pedestrians (2.7%). The proportion of pregnant women involved in MVCs as drivers exceeded 80% in all regions and different levels of urbanization, except the northern region and metropolitan areas, where the proportion of pregnant women as drivers was slightly lower (both 76.4%). The 18–24 age group had a higher proportion of passengers (29.9%) compared with older victims (40 + years old, 10.4%). Additionally, more pregnant women were involved in MVCs as passengers and pedestrians in the northern region (passengers: 19.0%; pedestrians: 4.6%) than in the southern region (passengers: 14.6%; pedestrians: 1.4%).

**Table 2:** Numbers and proportions of maternal motor vehicle crashes (MVCs) during pregnancy between 2008 and 2017 in Taiwan, according to vehicle type and different roles of road users

	Overall	Vehicle type			*p* value	Role of road users			*p* value
		Car	Two-wheeled motor vehicle	Others		Drivers	Passengers	Pedestrians	
	*N*	*n* (%)	*n* (%)	*n* (%)		*n* (%)	*n *(%)	*n* (%)	
Total	22134	7331 (33.1)	10604 (47.9)	4199 (19.0)		18024 (81.4)	3517 (15.9)	593 (2.7)	
*Maternal age at delivery (years)*									
18–24	3203	410 (12.8)	1770 (55.3)	1023 (31.9)	< 0.001	2186 (68.2)	959 (29.9)	58 (1.8)	< 0.001
25–29	5927	1596 (26.9)	3118 (52.6)	1213 (20.5)		4734 (79.9)	1033 (17.4)	160 (2.7)	
30–34	8146	3172 (38.9)	3732 (45.8)	1242 (15.2)		6934 (85.1)	997 (12.2)	215 (2.6)	
35–39	4105	1808 (44.0)	1690 (41.2)	607 (14.8)		3524 (85.8)	450 (11.0)	131 (3.2)	
40 +	753	345 (45.8)	294 (39.0)	114 (15.1)		646 (85.8)	78 (10.4)	29 (3.9)	
Mean ± SD	30.41 ± 5.20	32.05 ± 4.52	29.85 ± 5.16	28.96 ± 5.67		30.76 ± 5.03	28.48 ± 5.64	31.28 ± 5.17	
*Calendar year of delivery*									
2008–09	3097	1063 (34.3)	1454 (46.9)	580 (18.7)	0.0480	2528 (81.6)	485 (15.7)	84 (2.7)	0.1253
2010–11	3456	1202 (34.8)	1631 (47.2)	623 (18.0)		2844 (82.3)	513 (14.8)	99 (2.9)	
2012–13	4514	1519 (33.7)	2165 (48.0)	830 (18.4)		3704 (82.1)	690 (15.3)	120 (2.7)	
2014–15	5459	1772 (32.5)	2615 (47.9)	1072 (19.6)		4407 (80.7)	925 (16.9)	127 (2.3)	
2016–17	5608	1775 (31.7)	2739 (48.8)	1094 (19.5)		4541 (81.0)	904 (16.1)	163 (2.9)	
*Geographical area of residence *^*a*^									
North	7792	2198 (28.2)	3721 (47.8)	1873 (24.0)	< 0.001	5952 (76.4)	1483 (19.0)	357 (4.6)	< 0.001
Central	7061	2694 (38.2)	3258 (46.1)	1109 (15.7)		5974 (84.6)	954 (13.5)	133 (1.9)	
South	6588	2165 (32.9)	3344 (50.8)	1079 (16.4)		5537 (84.0)	962 (14.6)	89 (1.4)	
East	624	257 (41.2)	246 (39.4)	121 (19.4)		508 (81.4)	104 (16.7)	12 (1.9)	
*Urbanization of residence *^*a*^									
Urban	7184	2359 (32.8)	3389 (47.2)	1436 (20.0)	< 0.001	5952 (76.4)	1483 (19.0)	357 (4.6)	< 0.001
Satellite	8072	2376 (29.4)	4089 (50.7)	1607 (19.9)		5974 (84.6)	954 (13.5)	133 (1.9)	
Rural	6595	2510 (38.1)	2976 (45.1)	1109 (16.8)		5537 (84.0)	962 (14.6)	89 (1.4)	

^a^The inconsistency observed between the total population number and the sum of individual variable populations can be attributed to missing information

**Fig. 1:**
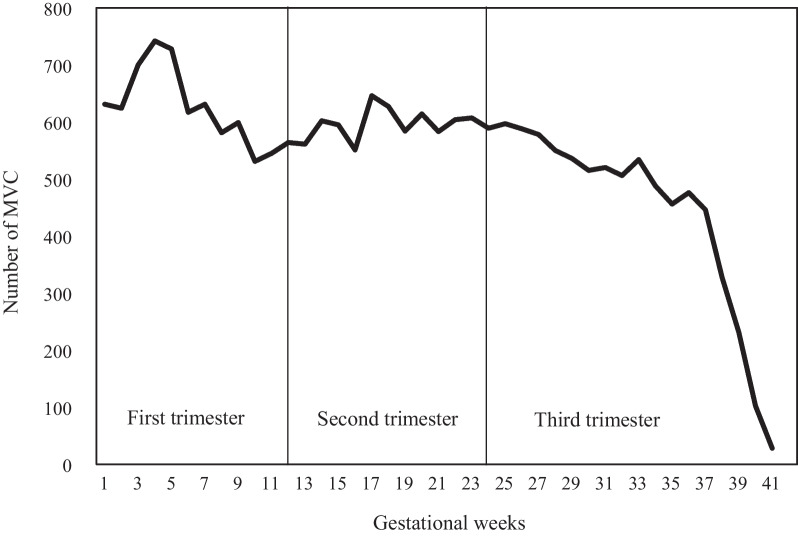
Gestational week specific numbers of MVC events during pregnancy in 2008–2017

Table 3 displays that the proportion of MVCs in each trimester was essentially similar. However, the proportion of pregnant women involved in car collisions increased in the third trimester (32.0%, 31.5%, and 36.5%), whereas two-wheeled motor vehicle-related incidents showed a decline as the trimesters progressed (36.6%, 33.0%, and 30.4%). Notably, the number of pregnant women involved in MVCs as passengers or pedestrians increased during the second and third trimesters. Figure 1 presents the number of MVC events according to gestational week.

**Table 3:** Overall and trimester-specific numbers and proportions of maternal MVCs during pregnancy between 2008 and 2017 in Taiwan, according to type of vehicle and role of road user. (*n* = 22,134)

	Overall	1st trimester	2nd trimester	3rd trimester	*p* value
	*N*	*n* (%)	*n *(%)	*n* (%)	
Total	22134	7541 (34.1)	7168 (32.4)	7425 (33.5)	
*Type of vehicle*					
Car	7331	2345 (32.0)	2310 (31.5)	2676 (36.5)	< 0.001
Two-wheeled motor vehicle	10,604	3880 (36.6)	3498 (33.0)	3226 (30.4)	
Others	4199	1316 (31.3)	1360 (32.4)	1523 (36.3)	
*Role of road users*					
Driver	18,024	6267 (34.8)	5829 (32.3)	5928 (32.9)	< 0.001
Passenger	3517	1107 (31.5)	1155 (32.8)	1255 (35.7)	
Pedestrian	593	167 (28.2)	184 (31.0)	242 (40.8)

**Fig. 2:**
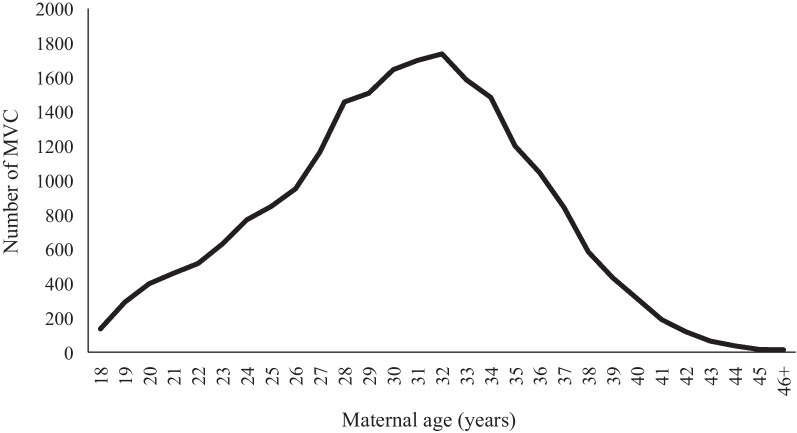
Maternal age specific numbers of MVC events during pregnancy in 2008–2017

The number of MVC events remained relatively consistent throughout pregnancy but decreased significantly after the 37th week of gestation (Fig. 1). This trend of a sharp drop after the 37th week of gestation was consistent across vehicle types and road user roles (Additional file 1: Fig. A1). Figure 2 demonstrates that the peak number of MVCs occurred at the maternal age of 32, and the number of events was high in the 28–34 age range. Additional file 1: Fig. A2 shows a similar pattern in terms of vehicle type, but the number of MVC events involving passengers and pedestrians remained stable across different maternal ages.

It is notable that our study also shows eastern residences have an almost twofold risk than northern after adjusting maternal age, calendar year at delivery, and urbanization of residence, from our additional regression analysis (Additional file 1: Table A1).

## Discussion

The prevalence rate of MVC among pregnant women in Taiwan from 2008 to 2017 was approximately 1.13%. It was mostly concentrated in pregnant women aged 28–34 and showed an increasing trend over the years. Prevalence involving young pregnant women and those residing in less urbanized areas was significantly high. Two-wheeled motor vehicle collisions accounted for the highest proportion of MVC cases involving pregnant women. Additionally, car and pedestrian collisions were concentrated in the third trimester.

The prevalence of crashes among pregnant women was 1.13%, which was found to be lower than the range reported in the previous studies conducted in the United States, which indicated that the risk ranges from 1.0 to 2.9% for pregnant women (Weiss & Strotmeyer 2002; Hyde et al. 2003; Schiff et al. 2010; Weiss et al. 2011; Vladutiu et al. 2013b). However, these studies employed different methodologies to estimate crash risk, and some of these differences are likely related to underlying differences in the population crash risk. Some studies assessed risk to drivers at MVCs, and others counted crashes that only occurred after 20th weeks of gestation. Direct comparison of crash risk might be a challenge because of these variations in approaches. One study conducted in Taiwan showed the crash rate of pregnant was obviously lower than the general population (1.28 vs. 1.85%) and scooter is the majority vehicle type of crashes (Chang et al. 2023), which findings are consistent with ours. We discovered that young pregnant women and those living in rural areas had a higher prevalence of MVCs. This finding aligned with previous studies showing that young drivers are at higher crash risk than older drivers (McCartt et al. 2009). One possible explanation for this age effect is the risky behavior often associated with young individuals, such as drinking or taking drugs. However, pregnant women typically reduce their exposure to such behaviors because of the potential adverse effects on maternal and fetal well-being. Another contributing factor to the elevated crash rate in young women may be lack of driving experience, sleep deprivation (Jou & Chao 2022; Groeger 2006), or simply because young women may travel more miles and take more trips than their older counterparts. The high crash risk in rural areas was supported by prior research (Zwerling et al. 2005). The remoteness of country roads leads to a decrease in police presence, which in turn increases the likelihood of speeding and phone use while driving (Truelove et al. 2023). Additionally, environmental factors, such as lack of lighting and poorly maintained roads, contribute to collisions in rural roads (López et al. 2016). The prevalence of MVCs increased over time which suggests that it may be related to the rising number of women holding driver’s licenses (Huang & Chung 2015) and the increase in women’s participation in the labor force (Department of gender equality, 2022). Furthermore, pregnant women aged 28–34 constituted the majority of crash victims, which is largely explained by more deliveries occurring in this age group. The large number of pregnant women affected by MVCs has important potential costs for the women themselves from injuries and loss of life, as well as potential burden to the healthcare system.

We observed that two-wheeled motor vehicle crashes accounted for the highest percentage of MVCs in pregnant women aged 18–24, and car crashes were most common in those aged 35 and above. This finding was consistent with a recent Taiwanese study that found an extremely large number of two-wheeled motor vehicle rider victims (aged 18–29). However, the number of car driver victims aged 30 or above was higher (Chang et al. 2022). The results reflected age-related preferences for different vehicles, but that might interact with socioeconomic status. For instance, the two-wheeled motor vehicle is a common mode of transportation for young individuals with limited budgets, which can contribute to the high prevalence of two-wheeled motor vehicle crashes among young pregnant women. Older pregnant women are more likely to drive cars and are therefore exposed to a higher car crash risk than younger ones. The percentages of two-wheeled motor vehicle crashes in pregnant women in the southern area (50.8%) and eastern area (41.2%) were higher than those in those in other areas. Challenges experienced by residents in eastern Taiwan in accessing medical resources are greater than those experienced by residents in the Western region (Chien et al. 2022). This discrepancy in accessibility potentially impacts travel distances, leading to variations in vehicle preferences. Given that two-wheeled motor vehicle is typically used for short-distance travel, and the issue of limited medical accessibility may influence individuals’ transportation choices. Our study revealed that the percentages of pregnant women involved in MVCs as passengers and pedestrians were higher in urban areas and the northern region of Taiwan than in other areas. This difference can be attributed to the well-developed public transportation system and the ease of travel without the need for driving in these areas.

A high number of car crash victims were observed in the third trimester, possibly because pregnant women often travel by car during the later stages of pregnancy. Conversely, pregnant women rarely use two-wheeled motors vehicle in the late stages of pregnancy, and thus, the proportion of two-wheeled motor vehicle crash victims was lower than that in the early stages of pregnancy. In terms of the roles of road users involved in MVCs, the proportion of passenger victims was high in the third trimester, whereas the proportion of car driver victims was high in the first trimester. The possible reason is that pregnant women who initially drove cars transitioned to being passengers in the later stages of pregnancy (Auriault et al. 2016). Notably, 40.8% of pedestrian victims were observed in the third trimester. A study investigating various factors related to pedestrian crashes found that being in proximity to schools, hospitals, and public transportation is associated with a high prevalence of pedestrian crashes (Rahman et al. 2022). Given that pregnant women have frequent visits to hospitals for prenatal care, particularly in the third trimester, this finding aligns with previous evidence and warrants further investigation.

One limitation of our study is that the database only included fetal records of pregnancies that lasted longer than 20 weeks. Any MVC that leads to early termination of pregnancy was not considered for analysis. Additionally, we lacked a real denominator (i.e., pregnant women who truly drove during the pregnancy), and thus determining the true exposure and frequency of travel of pregnant women were difficult. Lastly, as per the "Regulations Governing Road Traffic Accidents" in Taiwan, there is no obligatory obligation to notify the authorities in the event of a traffic accident, when there are no casualties. While the traffic collision data registry in Taiwan encompasses most motor vehicle collisions for insurance purposes, there is a potential for the registry to have insufficiently recorded minor motor vehicle collisions that resulted in no injuries or property damage. However, the advantage of our study is that we utilized nationwide police traffic collision data to describe the crash characteristics of pregnant women, providing a comprehensive overview of crash profiles during pregnancy in Taiwan. The large scale of MVC records of pregnant women allowed detailed analyses according to vehicle type, role of road user, and trimester. The use of nationwide data further eliminated potential selection biases.

## Conclusion

The young mothers contribute the highest proportion among pregnant MVCs victims. These findings from our study highlighted the importance of educating pregnant women between the ages of 28 and 34 regarding traffic collision prevention during perinatal care. Moreover, despite variations among different regions and in levels of urbanization, two-wheeled motor vehicle crashes accounted for nearly half of all MVCs involving pregnant women in Taiwan. Information from the current study can be valuable for the development of practical and effective strategies for preventing traffic collisions in Asian countries, where motorcycles are the main commute vehicles. This finding underscores the urgent need for targeted intervention strategies to reduce two-wheeled motor vehicle-related collisions.

Given the high risk to pregnant women, especially in their third trimester when they frequently visit clinics, efforts to enhance the safety design of sidewalks and roads to benefit all pedestrians are essential. For example, institutions ensure enough room to walk on the sidewalk near the clinic. They can also improve visibility by lighting up walkways in the street or increasing the available time for safe crossing. Additionally, setting up speed bumps on streets close to hospitals reduces the probability of traffic injury during patient visits for usual care. This comprehensive approach is crucial for safeguarding pregnant women and pedestrians alike. Future research should include an evaluation of efforts to reduce the risk of maternal traffic injuries.

### Supplementary Information

**Additional file 1.** Maternal characteristics supplementary tables and figures.

## Data Availability

The National Health Insurance Research Database (NHIRD) abides by data protection regulations in Taiwan, so the data in this study cannot be shared.

## Abbreviations

BN: Birth notification
MVCs: Motor vehicle crashes
