# Supplementary material for: Profiles of pregnant women encountering motor vehicle crashes in Taiwan, 2008–2017
Source: Inj Epidemiol. 2023 Dec 19;10:68. doi: 10.1186/s40621-023-00478-x (PMC10731885; doi:10.1186/s40621-023-00478-x)

**Supplemental table**

Supplemental table A1. The odds ratio of MVC occurrence in relation to maternal characteristics.

**Supplemental figure**

Supplemental Figure A1. Gestational week specific numbers of MVC events during pregnancy according to (a) type of vehicle and (b) role of road user.

Supplemental Figure A2. Maternal age specific numbers of MVC events during pregnancy according to (a) type of vehicle and (b) road of user.

Supplemental Figure A3. The four major (north, central, south, and east) geographic regions of Taiwan.

Supplemental table A1. The odds ratio of MVC occurrence in relation to maternal characteristics.

|  | *Crude OR* | *Adjusted OR* |
| --- | --- | --- |
| Maternal age at delivery (years) |  |  |
| 18-24 | 1.76 (1.68-1.83) | 1.74 (1.67-1.82) |
| 25-29 | 1.12 (1.08-1.16) | 1.13 (1.09-1.17) |
| 30-34 | 1.00 | 1.00 |
| 35-39 | 1.00 (0.97-1.04) | 0.98 (0.95-1.02) |
| 40+ | 1.05 (0.98-1.14) | 1.03 (0.96-1.11) |
| Calendar year of delivery |  |  |
| 2008-09 | 1.00 | 1.00 |
| 2010-11 | 1.19 (1.14-1.25) | 1.23 (1.17-1.29) |
| 2012-13 | 1.33 (1.27-1.39) | 1.38 (1.32-1.45) |
| 2014-15 | 1.63 (1.56-1.70) | 1.70 (1.62-1.77) |
| 2016-17 | 1.77 (1.69-1.85) | 1.84 (1.76-1.93) |
| Geographical area of ​​residence |  |  |
| North | 1.00 | 1.00 |
| Central | 1.75 (1.70-1.81) | 1.81 (1.74-1.88) |
| South | 1.69 (1.64-1.75) | 1.72 (1.66-1.78) |
| East | 1.88 (1.73-2.04) | 1.95 (1.78-2.13) |
| Urbanization of ​​residence |  |  |
| Urban | 1.00 | 1.00 |
| Satellite | 0.99 (0.96-1.02) | 0.93 (0.89-0.96) |
| Rural | 1.23 (1.19-1.27) | 1.05 (1.02-1.09) |

| (a) | (b) |
| --- | --- |

Supplemental Figure A1. Gestational week specific numbers of MVC events during pregnancy according to (a) type of vehicle and (b) role of road user.

| (a) | (b) |
| --- | --- |

Supplemental Figure A2. Maternal age specific numbers of MVC events during pregnancy according to (a) type of vehicle and (b) road of user.

Supplemental Figure A3. The four major (north, central, south, and east) geographic regions of Taiwan.


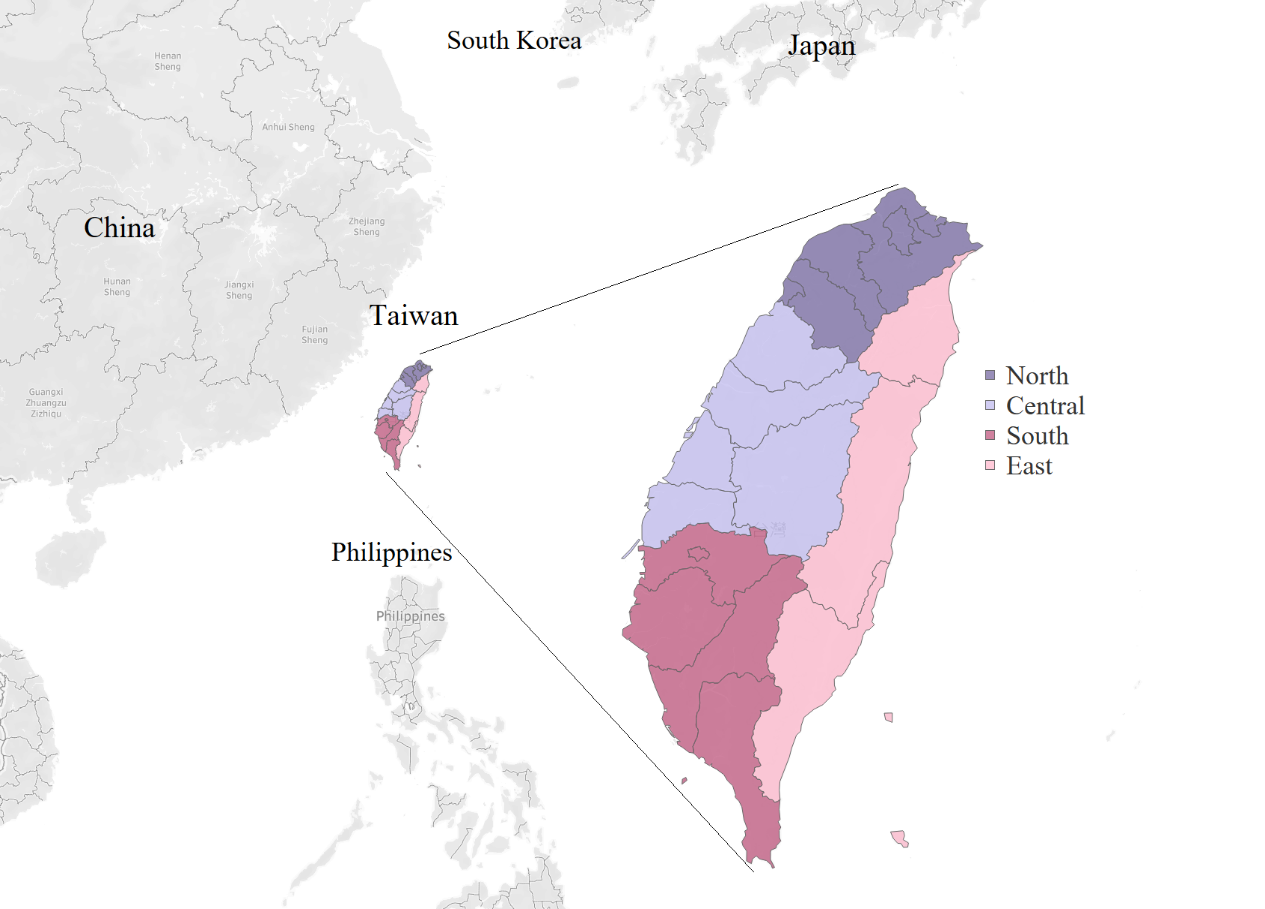

Supplement: Supplementary file 1 — Additional file 1. Maternal characteristics supplementary tables and figures. [file 40621_2023_478_MOESM1_ESM.docx]
